# *Porcine enteric alphacoronavirus* Inhibits *IFN-*α, *IFN-*β, *OAS, Mx1*, and *PKR* mRNA Expression in Infected Peyer's Patches *in vivo*

**DOI:** 10.3389/fvets.2020.00449

**Published:** 2020-07-03

**Authors:** Zhichao Xu, Lang Gong, Peng Peng, Yufang Liu, Chunyi Xue, Yongchang Cao

**Affiliations:** ^1^State Key Laboratory of Biocontrol, School of Life Science, Sun Yat-Sen University, Guangzhou, China; ^2^College of Veterinary Medicine, South China Agricultural University, Guangzhou, China

**Keywords:** coated *Porcine enteric alphacoronavirus* (PEAV)-loaded microspheres, pathogenesis, antiviral response, Peyer's patches, weaned piglets

## Abstract

*Porcine enteric alphacoronavirus* (PEAV) is a newly identified swine enteropathogenic coronavirus that causes watery diarrhea in neonatal piglets. The pathogenesis and host immune responses of PEAV infection are not fully characterized. The reason lies in the stomach environment, which would degrade cell-cultured live viruses *via* oral infection, making it difficult to establish an effective infection model to study the pathogenesis and host immune responses in pigs with a mature immune system. To solve this problem, in this study, coated PEAV-loaded microspheres were developed by centrifugal granulation-fluidized bed coating and demonstrated as an effective oral delivery system/animal infection model to protect PEAV virion against the complex gastrointestinal environment *in vitro* and to cause infection in weaned piglets *in vivo*. Weaned piglets orally inoculated with coated PEAV-loaded microspheres developed diarrhea and virus RNA was detected in rectal swabs from one to seven days post inoculation. In addition, microscopic lesions in the small intestine were observed, and viral antigens were also detected in the small intestines with PEAV immunohistochemical staining. Importantly, PEAV significantly inhibited mRNA expression of *IFN-*α, *IFN-*β, *OAS, Mx1*, and *PKR*, the genes involved in modulation of the host immune responses, in infected Peyer's patches, indicating that PEAV can overcome the antiviral response to cause damage when infection occurs. Collectively, our research successfully established a PEAV animal infection model in weaned piglets and suggested that the observed gene expression profile might help explain immunological changes associated with PEAV infection.

## Introduction

PEAV, as the newest strain of porcine CoVs, was first detected by our team via genomic analysis of samples collected from a diarrhea-outbreak in swine herds in Guangdong, China in 2017 (1), and this novel swine enteric CoV was subsequently confirmed to have existed in China since at least August 2016 by a retrospective detection study (2). In addition, the retrospective investigation of 236 samples from 45 swine farms showed that the prevalence of PEAV in pigs was reported to be 43.53% in Guangdong, China in August 2016 to May 2017 (2). Apart from Guangdong, a PEAV-like strain, CN/FJWT/2018, was recently discovered in Fujian, China (3), indicating a continuing threat to pig farms.

The complete genome of the PEAV strain GDS04 was reported after it was first identified (1). The full-length genome of PEAV is about 27 kb (1), arranged in the order of: 5′ UTR-ORF1a/1b-S-NS3-E-M-N-NS7a-3′ UTR (4). It is known that the S protein has many important characteristics in CoVs, such as virus attachment and entry, and induction of neutralizing antibodies *in vivo* (5). Of note, compare to other reported CoVs, PEAV has the smallest S protein of only 1129 amino acids (1).

PEAV caused clinical symptoms similar to other porcine enteric pathogens, such as PEDV and TGEV, characterized by vomiting, diarrhea, dehydration, and a mortality rate as high as 90% in piglets (1, 6). Since PEAV was reported in pigs (1), other groups have identified another two swine enteric HKU2-related CoVs, SADS-CoV, and SeACoV, in the same region. The newborn piglets were subsequently inoculated with isolated SADS-CoV and SeACoV strains which caused PEAV-like diarrheal disease (6–8).

PEAV is an important enteropathogen in pigs, but currently no report is involved in modulation of the host immune responses against PEAV infection. Oral infection of pigs with a mature immune system, like weaned piglets, can truly reflect the effect of the virus on the host immune responses. Moreover, according to our pre-experiment, cell-cultured PEAV doesn't cause infection in weaned piglets by orally feeding. In order to solve the problem, we successfully established an animal infection model of coated PEAV-loaded microspheres. In brief, we initially generated coated PEAV-loaded microspheres and evaluated the acid-resistance and enteric solubility *in vitro* and *in vivo* and further challenged microspheres to investigate the pathogenicity of PEAV in 33-day-old conventionally weaned piglets. Subsequent to the establishment of the animal infection model, we assessed the effect of PEAV on the antiviral molecules in Peyer's patches in inoculated piglets by real-time PCR.

## Materials and Methods

### Virus Propagation in Vero Cells

Vero cells were obtained from ATCC (ATCC number: CCL-81) (USA) and cultured in Dulbecco's modified eagle medium (DMEM) (Hyclone, USA), supplemented with 10% fetal bovine serum (FBS) (BOVOGEN, Australia), 100 U/mL penicillin, and 100 U/mL streptomycin in 37°C with 5% CO_2_ incubator. The maintenance medium for PEAV propagation was DMEM supplemented with 10 μg/mL trypsin (Gibco, USA) and cultured under the conditions described above. The PEAV GDS04 strain was isolated from piglets with severe diarrhea in our laboratory (8).

Virus propagation was performed as previously described (8). Briefly, Vero cells were seeded into T175 flasks and cultured for 90% confluence. One mL of PEAV GDS04 strain together with 50 mL of maintenance medium were added to flasks after the cell monolayers were washed three times with sterile pH 7.4 1 × phosphate buffered saline (PBS). The virus-inoculated cells were cultured continuously at 37°C in 5% CO_2_ to observe the cytopathic effect (CPE). Around one day post-infection (d.p.i.), >80% CPE was evidently observed in the inoculated cell monolayers; the flasks were then twice frozen at −80°C and thawed. The cells and supernatants were harvested together and stored at −80°C until subsequent viral titers' determination. Virus titers were calculated using the Reed-Muench method (9) and expressed as TCID_50_ per milliliter.

### Pigs

The animal study was approved by the Institutional Animal Care and Use Committee of the Sun Yat-sen University (Guangdong, China) and animals were treated in accordance with the regulations and guidelines of this committee. Thirty-three-day-old healthy conventional weaned piglets, crossbred of Duroc × Landrace × Native pigs of Guangdong of China, were procured from Wen' s Foodstuffs Group Co., Ltd. (Guangdong, China). All pigs were housed in the vivarium under standard environmental conditions and maintained in our animal facility with food and water *ad libitum* for a minimum of 7 days before the experimentation.

### Production of Coated PEAV-Loaded Microspheres (PEAV-Coated)

PEAV-Coated were prepared using centrifugal granulation- fluidized bed coating technology as described previously, with some modifications (10). Briefly, we added the commercial sucrose microspheres (450 g) (Anhui Sunhere Pharmaceutical Excipients Co., Ltd, China) into a centrifugal granulator (Shenzhen Xinyite Science and Technology Co., Ltd, China), and adjusted the solid rotation disc' s speed to 27 × *g* to keep the vorticity of the microspheres. We also adjusted the supply air velocity and the exhaust air velocity to 326 × *g* and 666 × *g*, respectively. We subsequently set the machine to spray pure water (80–100 mL) to blend the powders into the microspheres at 0.02 × *g* for 30 min by a tangential spray nozzle. About 136 g new powders were constantly added into the microspheres. Then the final product was dried at 37°C for 1 h under the same air quantity. Subsequently, the dried PEAV-loaded microspheres smaller than 0.7 mm were screened by sieving, and then filled into a fluidized bed apparatus with a bottom spray configuration (Shenzhen Xinyite Science and Technology Co., Ltd., China). We used the magnetic stirrer to mix enteric coating suspensions (Weight gain 25%) with 6% Hydroxypropyl methyl cellulose phthalate (HPMCP) (Shanghai Yunhong Chemical Co., Ltd., China), and then PEAV-loaded microspheres were continuously sprayed with the enteric coating suspensions at 0.25 mL/min through a spray nozzle at the bottom of the fluidized bed apparatus. Of note, the relative bed temperature needed to be kept at 29°C to avoid an agglomeration of the microspheres during the coating process. After all the enteric coating suspensions were sprayed out, the products needed to dry for an additional 30 min.

### Characterization of PEAV-Coated

According to the method described by Kashappa-Goud H. Desai and Steven P. Schwendeman (11), we used a Hitachi S-3400N scanning electron microscope (Hitachi, Japan) to examine the surface morphology of microspheres by taking SEM images. Briefly, we fixed the microspheres on a brass stub with a double-sided adhesive tape and then coated them with ~3–5 nm electrically conductive gold for 100 s at 40 W in a vacuum. Then, the excitation voltage was set as 8–10 kV to take the images of the microsphere surface. One hundred randomly selected samples of PEAV-Coated were selected to measure the diameter with a Vernier caliper (Guangzhou Heyue Biotechnology Co., Ltd., China) to determine the size distribution of the PEAV-Coated. In addition, 100 samples of PEAV-Coated or sucrose microspheres were randomly selected and measured with Electronic scales (Sartorius Group, Germany) to calculate the weight gain of the single microsphere and to analyze the weight gain of the single PEAV-Coated.

### *In vitro* and *in vivo* Acid Resistance and Enteric Solubility Study of PEAV-Coated

In line with previous studies (12), we used simulated gastric fluid and simulated intestinal fluid to determine the acid resistance and enteric solubility of PEAV-Coated *in vitro*. Briefly, PEAV-Coated were successively incubated in pH 1.2 simulated gastric fluid prepared with 2 g/L NaCl, 3.2 g/L porcine pepsin, 0.7% HCl, and pH 6.8 simulated intestinal fluid prepared with 6.8 g/L NaH_2_PO_4_, 7.7% 0.2 N NaOH, and 10 g/L pancreatin at 37°C for 2 h, and freeze-dried powders containing the PEAV GDS04 strain were treated under the same conditions. The TCID_50_ assay as described above was used to analyze the acid resistance of PEAV-Coated after the simulated gastric fluid treatment.

To analyze the acid resistance and enteric solubility of PEAV-Coated *in vivo*, weaned piglets were orally inoculated with these microspheres to assess virus shedding according to previous studies with some modifications (10). Briefly, 18 conventionally weaned piglets were randomly divided into three groups with six piglets in each, and were housed in three separate rooms. On day 0, weaned piglets in group one were orally challenged with 20 g of PEAV-Coated containing a total of 2 × 10^5^ TCID_50_ of the PEAV GDS04 strain (1 gram of microspheres contained 1 × 10^4^ TCID_50_ of PEAV GDS04 strain). Weaned piglets in groups two and three as controls were orally inoculated with 20 mL of maintenance medium or 20 mL of maintenance medium containing a total of 2 × 10^5^ TCID_50_ of the PEAV GDS04 strain (1 milliliter of medium contained 1 × 10^4^ TCID_50_ of PEAV GDS04 strain), respectively. After challenge, rectal swabs were collected from each piglet on day 0, 2, 4, 6, and 8 after the challenge to assess PEAV virus shedding with real-time PCR as described below.

### Experimental Infection With PEAV-Coated in Conventional Weaned Piglets

Twenty-four conventionally weaned piglets, negative of the major porcine enteric viruses including PDCoV, PEDV, TGEV, PRoV, and PEAV by testing the rectal swabs on day −1 as previously described (13), were randomly divided into two groups with 12 piglets in each and were housed in two separate rooms. On day 0, weaned piglets in one group were orally challenged with 100 g/head of PEAV-Coated containing a total of 1 × 10^6^ TCID_50_ of the PEAV GDS04 strain (1 gram of microspheres contained 1 × 10^4^ TCID_50_ of PEAV GDS04 strain) for 3 days and weaned piglets in another group were orally inoculated with 100 g/head sucrose microspheres for 3 days and served as uninfected controls. After infection, clinical signs of vomiting, diarrhea, and lethargy were observed daily in each piglet. In addition, the diarrhea severity of each piglet was scored daily according to the previous criteria (14): 0 = normal, 1 = soft (cowpie), 2 = liquid with some solid content, 3 = watery with no solid content.

Rectal swabs were collected from each piglet before inoculation and then every day until 7 d.p.i. and were homogenized in 1 mL sterile pH 7.4 1 × PBS immediately after collection. Six of the challenged piglets and six of the negative control piglets were randomly selected from each group and humanely sacrificed for necropsy at 3 d.p.i., and the remaining weaned piglets were necropsied at 7 d.p.i. At necropsy, the fresh Peyer's patches from ileum were collected for analysis of the antiviral molecules with real-time RT-PCR and the fresh jejunum were also analyzed with histopathology and immunohistochemistry.

### RNA Isolation and Real-Time PCR Analysis

RNA extraction and RT were performed as previously described with some modifications (8). Briefly, viral RNA was extracted from the rectal swab fluids from each piglet by using an RNeasy kit (Magen, China) according to the manufacturer' instruction. Two μg of viral RNA was converted to cDNA by using an RT-PCR kit (TaKaRa, Dalian). Primers for the nucleocapsid (*n*) gene of PEAV (sense: 5′-CTGACTGTTGTTGAGGTTAC-3′; antisense: 5′-TCTGCCAAAGCTTGTTTAAC-3′), and probe (5′-FAM-TCACAGTCTCGTTCTCGCAATCA-TARMA-3′) were designed as previously described (15) and synthesized by Invitrogen Company (Shanghai, China). The real-time PCR assay was performed on an Applied Biosystem 7500 instrument (Life Technologies, USA) with a 20-μL volume containing 1 μL of cDNA, 10 μL of Thunderbird Probe qPCR Mix, 0.04 μL 50 × Rox reference dye (TOYOBO, Shanghai), 0.2 μmol/L of probe, and a 0.3 μmol/L of each gene-specific primer. The PCR program was as follows: 95°C for 30 s; 45 cycles of 95°C for 5 s, 62°C for 30 s. The *n* gene was amplified by using the specific primers (sense: 5′-CCGCTCGAGATGGCAACTGTTAATTGG-3′; antisense: 5′-CGCGGATCCCGATTAATAATCTCATCCAC-3′) that were designed according to the sequence of PEAV strain GDS04 (GenBank, Accession no: MF167434.1), and the PCR products were ligated with the pEGFP-N1 vector (Clontech, USA) by using a PCR cloning kit (NEB, USA), and then the 10-fold serially diluted known plasmid concentration was used as the template to construct a real-time PCR standard curve in each plate. The quantity of PEAV viral RNA in rectal swabs was calculated based on the cycle threshold (Ct) values for the standard curve.

To analyze antiviral molecular changes in the piglets with PEAV infection, equal quantities (1 g) of Peyer's patches were homogenized in sterile pH 7.4 1 × PBS, and 200 μL of the supernatant was used for RNA extraction by using an RNeasy kit (Magen, China) following the manufacturer's instruction. Two μg of total RNA was converted to cDNA by using an RT-PCR kit (TaKaRa, Dalian). The specific primers for porcine *IFN-*α (sense: 5′-TCTCATGCACCAGAGCCA-3′; antisense: 5′-CCTGGACCACAGAAGGGA-3′), *IFN-*β (sense: 5′-AGTGCATCCTCCAAATCGCT-3′; antisense: 5′-GCTCATGGAAAGAGCTGTGGT-3′), *PKR* (sense: 5′-AAAGCGGACAAGTCGAAAGG-3′; antisense: 5′-TCCACTTCATTTCCATAGTCTTCTGA-3′), *OAS* (sense: 5′-GAGCTGCAGCGAGACTTCCT-3′; antisense: 5′-TGCTTGACAAGGCGGATGA-3′), *Mx1* (sense: 5′-GGCGTGGGAATCAGTCATG-3′; antisense: 5′-AGGAAGGTCTATGAGGGTCAGATCT-3′), and glyceraldehydes-3-phosphate dehydrogenase (*GAPDH*; sense: 5′-CCTTCCGTGTCCCTACTGCCAAC-3′; antisense: 5′-GACGCCTGCTTCACCACCTTCT-3′) were designed as previously described (16, 17) and synthesized by Sangon Company (Shanghai, China). The real-time PCR assay was performed on an Applied Biosystem 7500 instrument (Life Technologies, USA) with a 20-μL volume containing 1 μL of cDNA, 10 μL of 2 × SYBR green Premix *Ex Taq* (TaKaRa, Dalian), and 0.4 μM of each gene-specific primer. The amplification conditions were referring to previous publications (16) and were as follows: 95°C for 30 s; then 40 cycles of 95°C for 3 s, 60°C for 30 s; and 1 cycle of 95°C for 15 s, 60°C for 1 min, and 95°C for 15 s, 60°C for 15 s. A melt curve for the PCR products was obtained to determine the specificity of the amplification at the final step. The antiviral molecules expressions were calculated relative to the expression of the reference gene *GAPDH* and presented as the change (*n*-fold) relative to the control samples.

### Histological and Immunohistochemical Staining

Histological and Immunohistochemical staining were performed as previously described with some modifications (8). Briefly, at necropsy, the jejunum tissue samples of the piglets from the challenged and control groups were separated, routinely fixed in 10% formalin, embedded, sectioned, and stained with hematoxylin and eosin (H&E); the slides were then examined and analyzed with conventional light microscopy. Five-μm sections of formalin-fixed paraffin-embedded tissues were mounted onto positively charged glass slides. Slides were air dried at 60 °C for 120 min prior to deparaffinization. Slides were then rinsed and incubated with target retrieval solution (Servicebio, China). The sections were incubated with PEAV (GenBank, Accession no: MF167434.1) specific mouse antisera (Wen's Foodstuffs Group Co., Ltd, China) (1:400) as the primary antibody for 12 h at 4 °C after to block with 1% BSA (Solarbio, China). They were then incubated with peroxidase-labeled goat anti-mouse IgG secondary antibody (Dako, Denmark) (1:200) for 50 min at room temperature prior to visualization with a 3, 3′-diaminobenzidine (DAB) chromogen kit (Dako, Denmark). In addition, hematoxylin was used for counterstaining. Jejunum tissue samples from uninfected piglets were used as a negative control.

### Statistical Analysis

Statistical comparisons were performed using GraphPad Prism software. The significance of the differences between the treatment group and controls in the mRNA expressions [TCID_50_, antiviral molecules (*IFN-*α*/*β, *OAS, PKR*, and *Mx1*)] was determined by the ANOVA and Mann-Whitney accordingly.

## Results

### Centrifugal Granulation-Fluidized Bed Coating Manufactures the PEAV-Coated

**Figure 1:**
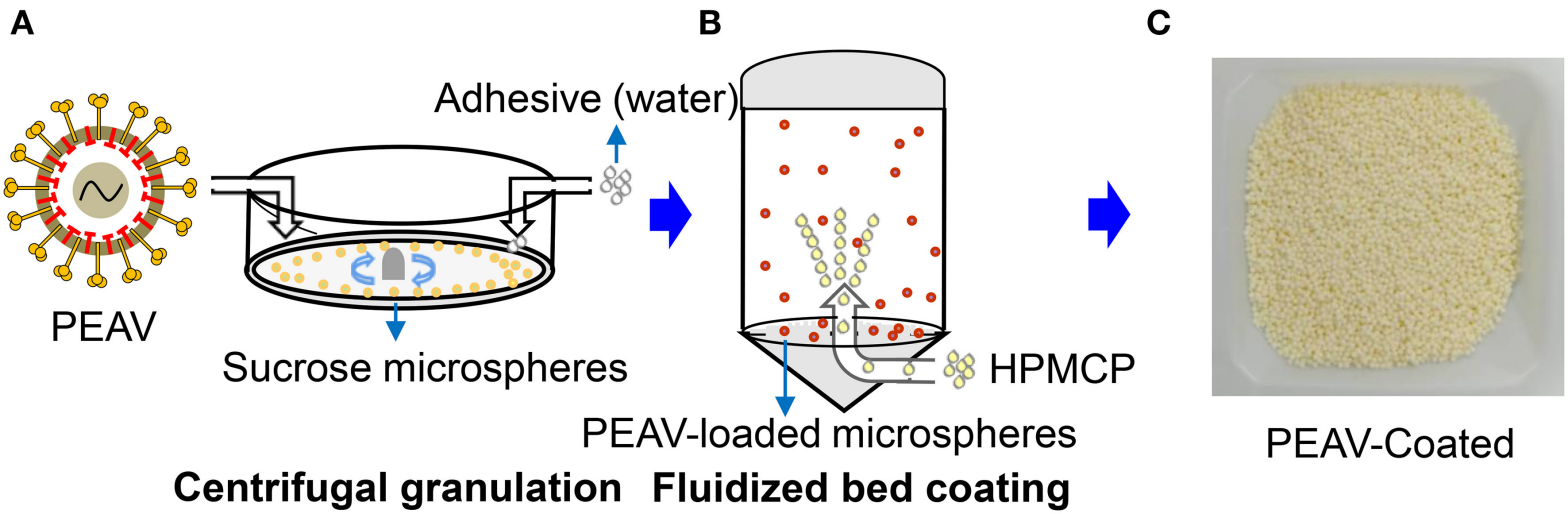
Generation of PEAV-Coated by Centrifugal granulation-fluidized bed coating. **(A)** The freeze-dried powders containing the PEAV GDS04 strain were loaded onto sucrose microspheres with pure water as an adhesive by using centrifugal granulation technology. The enteric coating (HPMCP) **(B)** was successively sprayed onto the PEAV-loaded microspheres by using a fluidized bed apparatus. **(C)** The final product was the PEAV-Coated.

Viruses by oral feeding failed to infect the body due to the labile components that were degraded by the acid pH of the stomach (18). To successfully infect the animals, we used centrifugal granulation technology to load the freeze-dried powders containing the PEAV GDS04 strain onto sucrose microspheres (Figure 1A). To protect PEAV from low pH while facilitating controlled virion release in the intestine, pH-resistant enteric coating (HPMCP) was loaded on the PEAV-loaded microspheres using a fluidized bed coating apparatus after a drying process (Figure 1B). The final products of the manufacturing process yielded the coated mini-spheres, with a faint-yellow surface (Figure 1C).

### PEAV-Coated Structural Characterization

**Figure 2:**
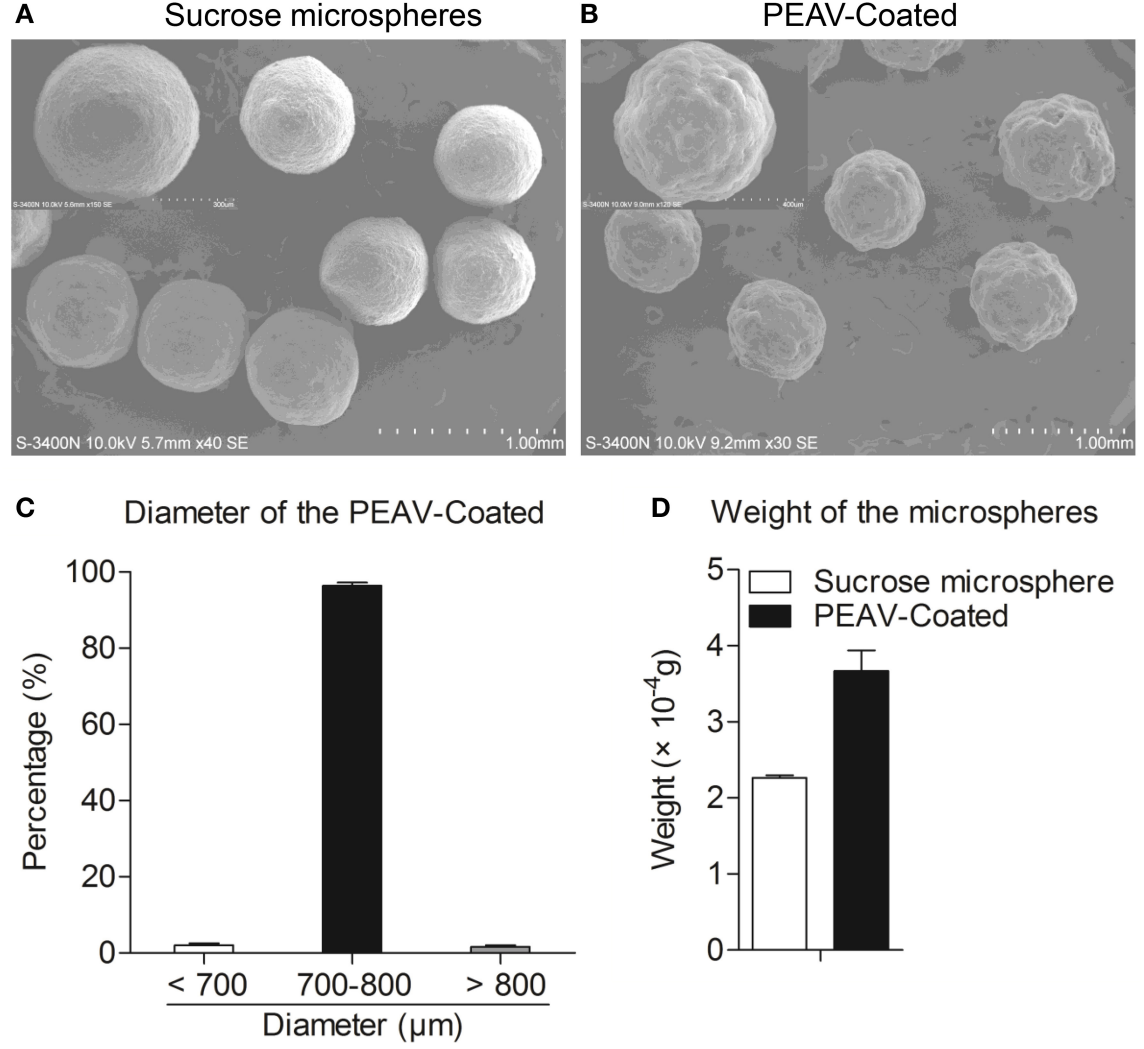
Appearance, size distribution, and weight gain of PEAV-Coated. Scanning electron micrographs of sucrose microspheres **(A)** and PEAV-Coated **(B)** (scale bar 1.0 mm, 300 μm or 400 μm in **A,B**). **(C)** Size distribution of freshly prepared PEAV-Coated was measured using a Vernier caliper and data were plotted as the percentage of different diameters. **(D)** One hundred freshly prepared PEAV-Coated or sucrose microspheres were randomly selected and measured with Electronic scales to calculate the weight gain of the single microsphere. Results are representative of three independent experiments. Data are represented as mean ± SD, *n* = 3.

To observe the encapsulation effect of PEAV in the microspheres, we used a scanning electron microscope to examine the morphology of the microspheres. As shown in Figure 2B, a scanning electron micrograph revealed the microparticles containing PEAV characterized by their pill shape, rough surface, lack of porosity, and relatively uniformed size. In addition, compared to sucrose microspheres, PEAV-Coated were around 1.3 times larger (Figure 2A). Further, we found that more than 95% of microspheres were in the size range of 700–800 μm in diameter (Figure 2C). In addition, single PEAV-Coated weight gained 60% more than single sucrose microspheres (Figure 2D).

### PEAV-Coated Have Acid Resistance and Enteric Solubility *in vitro* and *in vivo*

**Figure 3:**
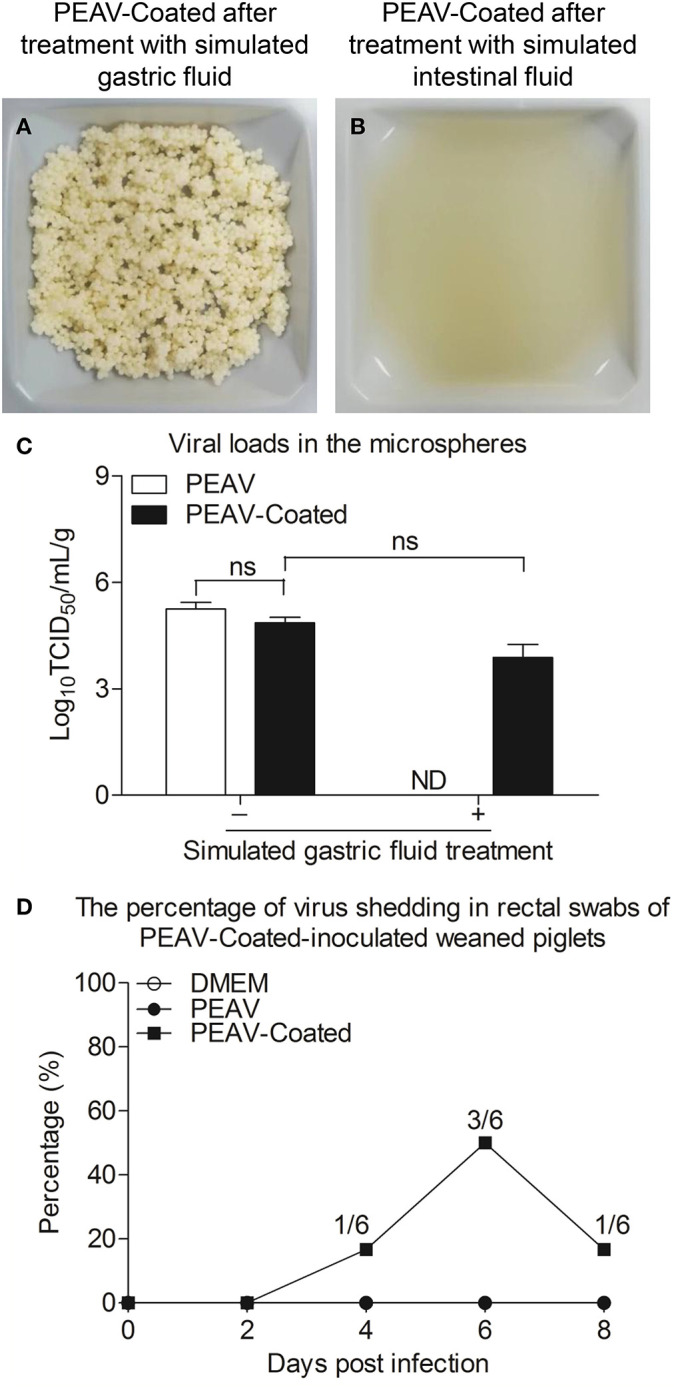
Acid resistance and dissolvability of the PEAV-Coated in intestinal fluid *in vitro* and *in vivo*. PEAV-Coated were treated with the simulated gastric fluid **(A)** and the simulated intestinal fluid **(B)**. The acid resistance of the PEAV-Coated was analyzed before and after the simulated gastric fluid treatment by a TCID_50_ assay, and PEAV was treated and analyzed equally as the control **(C)**. Weaned piglets were orally inoculated with PEAV-Coated, PEAV, and maintenance medium. Rectal swabs were collected from the piglets on days 0, 2, 4, 6, and 8 post inoculation and the virus shedding of PEAV were examined through real-time PCR using specific primers. The percentage of PEAV infection was calculated in **(D)**. Results of TCID_50_ are representative of three independent experiments. Data are represented as mean ± SD, *n* = 3. ND means non-detectable.

Considering that viruses by oral feeding overcoming the acid pH of the stomach is the key to infection, we examined the acid resistance and enteric solubility of PEAV-Coated *in vitro* and *in vivo*. As shown in Figures 3A,B, we found that the shape of PEAV-Coated was unaffected after simulated gastric fluid treatment, but it could dissolve in simulated intestinal fluid. In addition, compared to the microspheres before treatment, virus titers in microspheres after the simulated gastric fluid treatment dropped slightly (Figure 3C). To further determine whether PEAV-Coated could resist gastric acid *in vivo*, we fed weaned piglets with PEAV-Coated and the control groups with DMEM without mixing with sucrose microspheres or PEAV without mixing with sucrose microspheres. As shown in Figure 3D, in half of the weaned piglets orally inoculated with PEAV-Coated PEAV RNA could be detected in fecal swabs at the sixth day after the challenge. In contrast, no PEAV RNA were detected in DMEM and PEAV-inoculated weaned piglets.

### PEAV-Coated Caused Diarrhea in Weaned Piglets

**Figure 4:**
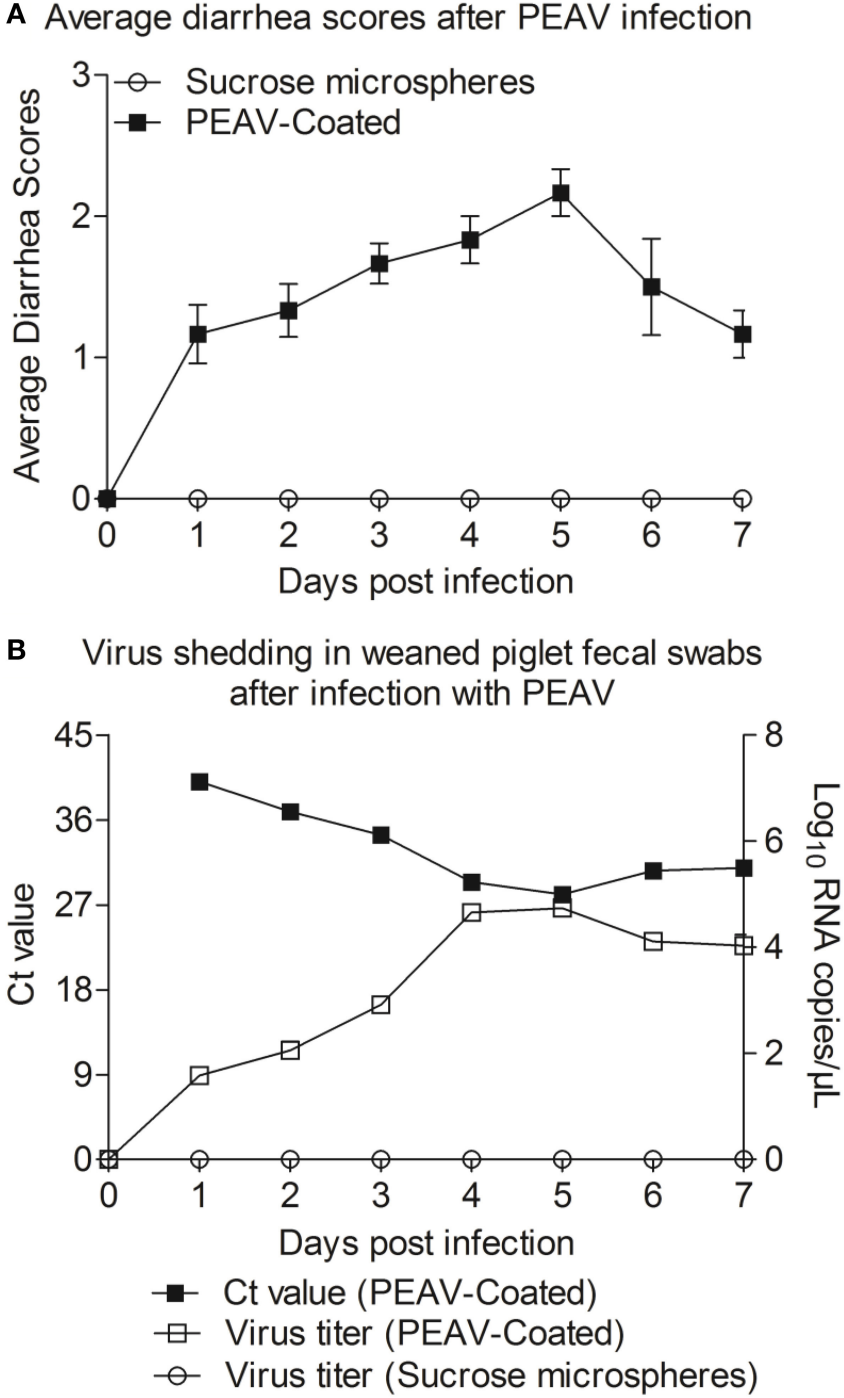
Reproduction of watery diarrhea and fecal viral shedding in weaned piglets inoculated with PEAV-Coated *via* oral feeding. **(A)** Average diarrhea scores after PEAV-Coated infection. **(B)** Ct values of group PEAV-Coated inoculated weaned piglet fecal swabs and viral RNA shedding in fecal swabs after PEAV-Coated inoculation or sucrose microspheres inoculation.

In order to determine whether PEAV-Coated could infect weaned piglets, 33-day old conventionally weaned piglets were orally infected with PEAV-Coated at a dose of 1 × 10^6^ TCID_50_/100 g/head. Compared to the negative control, watery diarrhea was observed in all weaned piglets inoculated with PEAV-Coated from 1 d.p.i. to 7 d.p.i. (Figure 4A). We further examined the viral shedding by real-time PCR in fecal swabs collected from inoculated-piglets from 1 d.p.i. to 7 d.p.i. As shown in Figure 4B, PEAV RNA was detected in PEAV-Coated-challenged piglets, while no PEAV RNA was detected in sucrose microspheres-challenged piglets during the study. Taken together, these results suggest that PEAV-Coated could infect weaned piglets to cause diarrhea *in vivo*.

### PEAV-Coated Inhibits an Antiviral Response in Peyer's Patches

**Figure 5:**
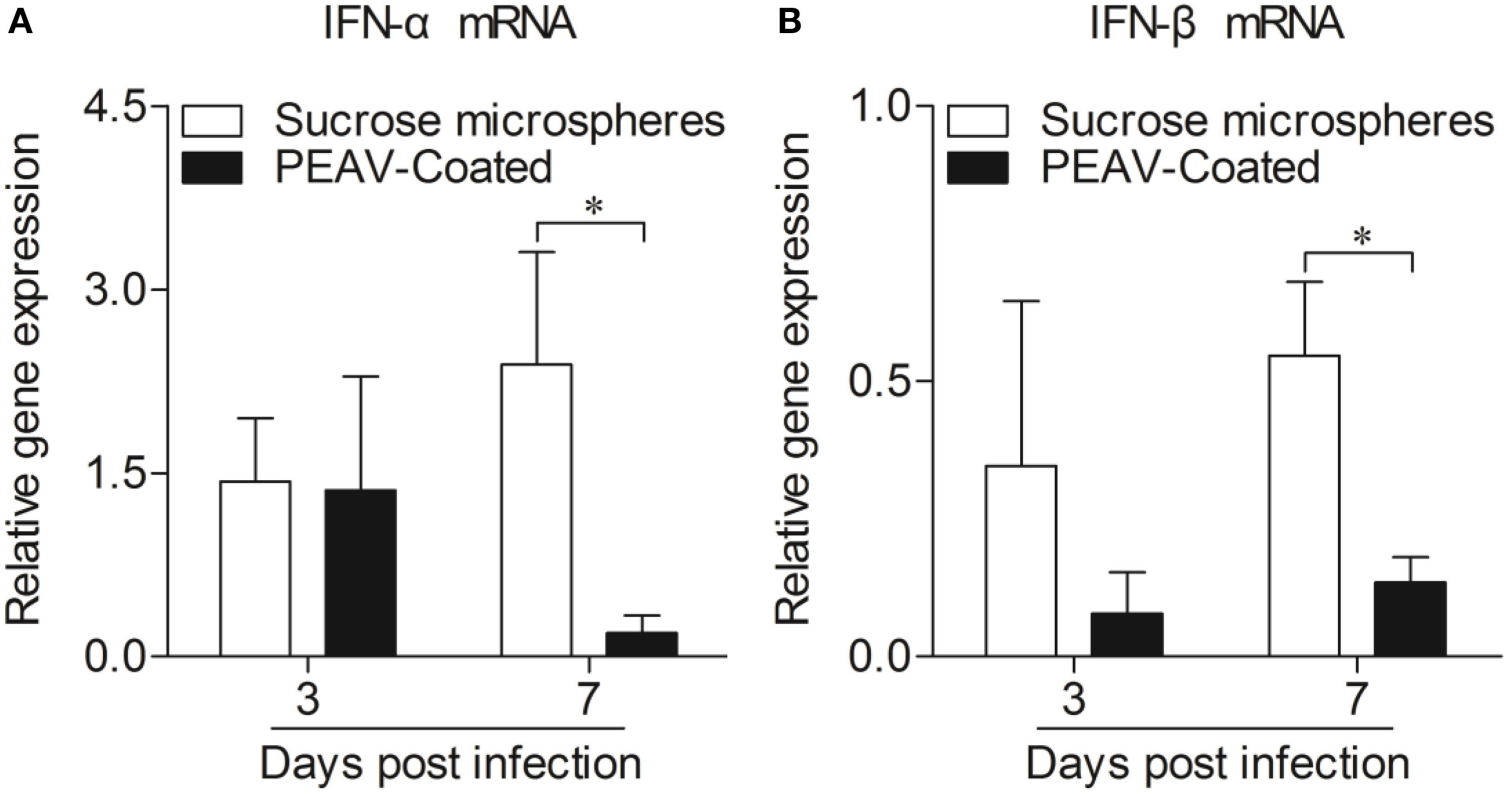
Expressions of mRNA of type I interferon in Peyer's patches from weaned piglets after infection with PEAV-Coated at 3 d.p.i. and 7 d.p.i. The mRNA expressions of *IFN-*α **(A)** and *IFN-*β **(B)** in Peyer's patches from weaned piglets after infection with PEAV-Coated were examined with real-time PCR using specific primers at 3 d.p.i. and 7 d.p.i. The mRNA expression levels of these cytokines were calculated relative to the expression level of *GAPDH*. Data are represented as mean ±SD, *n* = 6. *p < 0.05.

**Figure 6:**
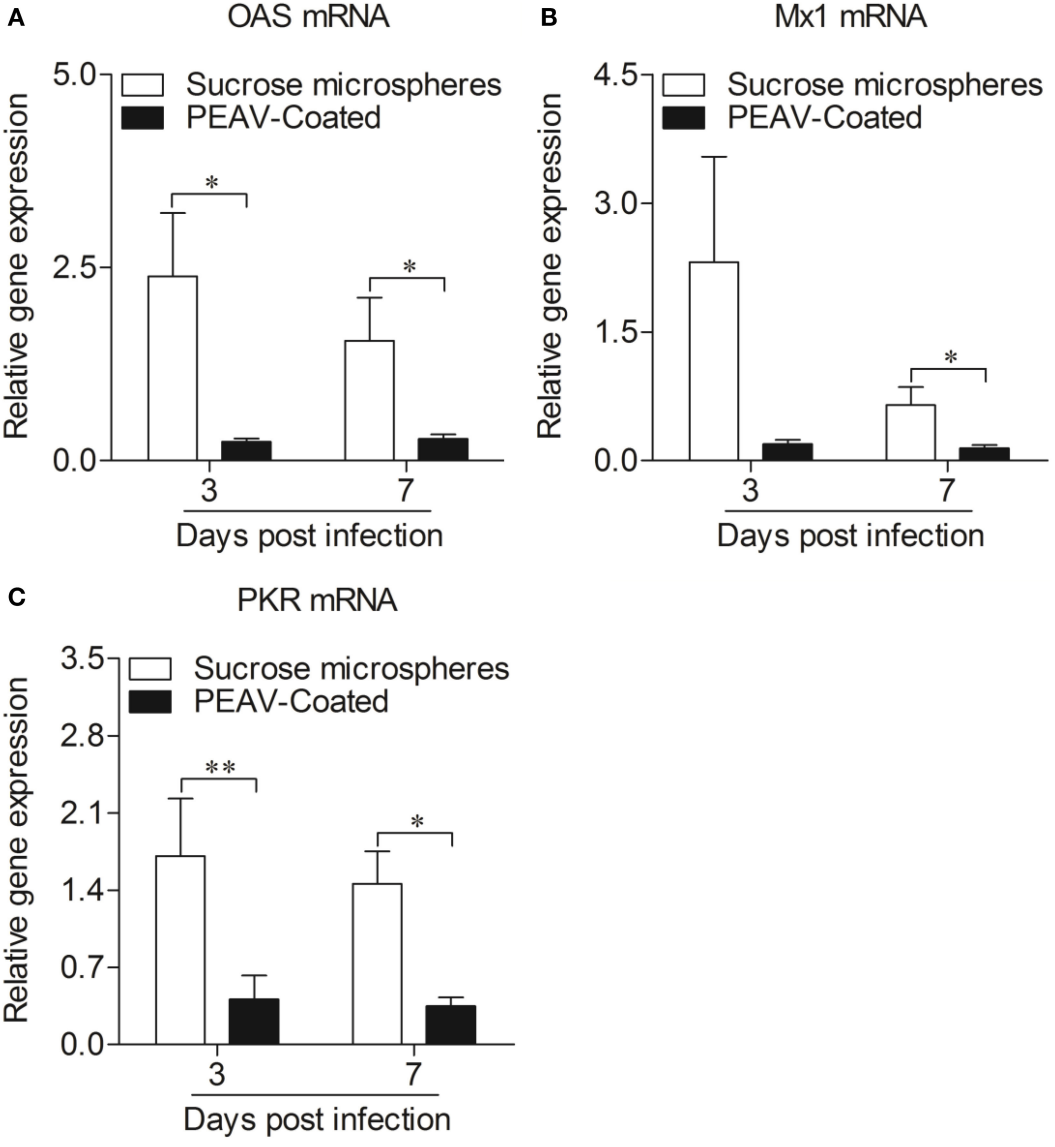
Expressions of antiviral molecules mRNA in Peyer's patches from weaned piglets after infection with PEAV-Coated at 3 d.p.i. and 7 d.p.i. The mRNA expressions of *OAS* **(A)**, *Mx1* **(B)**, and *PKR* **(C)** in Peyer's patches from weaned piglets after infection with PEAV-Coated were examined with real-time PCR using specific primers at 3 d.p.i. and 7 d.p.i. The mRNA expression levels of these molecules were calculated relative to the expression level of *GAPDH*. Data are represented as mean ± SD, *n* = 6. **p < 0.01, *p < 0.05.

Type I interferon (IFN-α/β) plays an important role in innate immune response, which prompted us to examine the effect of PEAV on IFN-α and IFN-β. We found that PEAV could inhibit the mRNA expressions of *IFN-*α (*p* < 0.05) and *IFN-*β (*p* < 0.05) in Peyer's patches from PEAV-Coated-challenged weaned piglets at 7 d.p.i. (Figure 5). It was reported that IFN-stimulated genes (ISGs) could be induced after IFNs production (19). We further examined the mRNA expressions of ISGs in Peyer's patches from weaned piglets infected with PEAV-Coated at 3 d.p.i. and 7 d.p.i. As shown in Figure 6, we found that PEAV could inhibit the mRNA expressions of *OAS* (*p* < 0.05), *Mx1* (*p* < 0.05), and *PKR* (*p* < 0.01 or *p* < 0.05) in Peyer's patches, indicating that PEAV could overcome an antiviral response to infect pigs.

### PEAV-Coated Caused Histopathological Lesions and had Viral Antigen Distribution in Small Intestine

Pathology tests were conducted to determine the histological changes in the jejunum of weaned piglets infected with the PEAV-Coated. Compare to the negative control (Supplemental Figures 1A,C), the typical histological lesions characterized by intestinal villus detachment due to injury of intestinal epithelial cells were observed in intestinal villus from piglets that were necropsied at 3 d.p.i. and 7 d.p.i. (Supplemental Figures 1B,D). Consistent with the histopathological results, PEAV antigens were detected in the villous enterocytes of jejunum collected from PEAV-Coated-challenged piglets that were necropsied at 3 d.p.i. and 7 d.p.i. (Supplemental Figures 1F,H), but no PEAV antigen in the negative control was detected by immunohistochemical analysis (Supplemental Figures 1E,G), indicating that PEAV-Coated could cause intestinal lesions in weaned piglets.

## Discussion

Since PEAV was first reported in pigs in early February 2017 in Guangdong, China (1), this novel swine enteric CoV has been widely detected in areas of southern China, including Guangdong and Fujian (2, 3). Although a few studies have demonstrated that PEAV was highly pathogenic to newborn piglets (6–8), there are no published papers reporting the pathogenicity of PEAV in weaned piglets and the effect of PEAV infection on antiviral responses *in vivo* is still unclear. In the present study, PEAV-Coated were developed by the centrifugal granulation-fluidized bed coating apparatus and were used on orally inoculated weaned piglets to evaluate the pathogenicity to weaned piglets and to set up a model to explore antiviral response *in vivo*.

Oral infection of pigs with mature immune systems, like weaned piglets, can truly reflect the effect of the enterovirus on the host's immune responses. In another of our studies, we found that weaned piglets inoculated with cell-cultured PEAV GDS04 strain at a medium dose (2 × 10^5^ TCID_50_) could not develop diarrhea and that no PEAV was detected in the rectal swabs (data not shown), which was possibly due to the fact that the viruses could not overcome the acid pH level of the stomach by oral feeding, which can degrade labile components (18). This contradicts the idea that PEAV is transmitted through the fecal-oral route (20). We speculated that on farms, weaned piglets with mature immune systems might be infected by feeding on PEAV-infected pigs' feces, which may protect the virus from stomach acid damage. This has been confirmed by the protective effect of feed-back in sows with intestinal contents containing PEDV (21). Since it has been reported that the PEDV oral vaccine prepared by centrifugal granulation-fluidized bed coating technology could protect PEDV antigens against the complex gastrointestinal environment *in vitro* and *in vivo* and induced obvious immune responses in weaned piglets (10), we proposed that oral pellets containing the virus prepared by centrifugal granulation-fluidized bed coating technology might overcome the low pH and enzymes of the stomach to infect the body. To test our hypothesis, freeze-dried powders containing the PEAV GDS04 strain were loaded onto sucrose microspheres using centrifugal granulation technology. It was reported that HPMCP can improve the digestive stability and intestinal transport of green tea catechins (22). To further facilitate PEAV release in the gut, HPMCP was selected as an enteric polymer by fluidized bed coating technology. As we discussed above, for infection in weaned piglets, gastric acid resisted by PEAV-Coated is the key. As shown in Figure 3, PEAV-Coated was verified to be able to resist acid in *in vitro* and *in vivo* experiments, while PEAV were to a large extent reduced by the simulated gastric acid treatment or no PEAV was detected in the rectal swabs from PEAV-inoculated weaned piglets. In addition, we found that there were no significant differences in virus titers between PEAV freeze-dried powders and PEAV-Coated, indicating that the virus survival was unaffected by the sucrose microspheres, the HPMCP, and the preparation process. This information suggests coated microspheres prepared by centrifugal granulation-coating technology might be a common and effective oral delivery system to protect the virus against the complex gastrointestinal environment to achieve infection *in vivo*. This is of great importance because it can be used to study the effect of enteroviruses on the host immune system *in vivo*. In addition, in order to control intestinal CoV infection in piglets, it requires viable virus particles to reach the intestine to generate mucosal immunity in sows, which is passed on to piglets *via* milk (23). Our research in PEAV prepared a candidate tool for the effective control of PEAV.

As a newly identified swine pathogen, the pathogenicity of PEAV in weaned piglets is still unknown. We infected the 33-day-old weaned piglets with the PEAV-Coated *via* oral feeding. While PEAV normally leads to severe watery diarrhea in newborn piglets (8), PEAV only caused mild diarrhea in weaned piglets, which suggests that the pathogenicity of PEAV varies among pigs of different ages, but it still poses a huge threat to weaned piglets and newborn piglets in pig farms. Interestingly, unlike in newborn piglets (6, 8), there was no vomiting and death in weaned piglets infected by PEAV-Coated (data not shown), which was found in other porcine enteric CoVs infection, such as PDCoV, PEDV, and TGEV (24–26), indicating that these CoVs are more harmful to newborn piglets than weaned piglets. However, compared to PEAV-Coated, cell-cultured PEAV without coated didn't cause any diarrhea in weaned piglets (data not shown). Taken together, all these results speculate that age-dependent pathogenicity in PEAV as well as in other entero-CoVs is related ti stomach acid degradation.

Neonatal suckling piglets are not appropriate targets to study immune responses by virus infection due to their fragile and immature immune systems (26). Weaned piglets were used to successfully reveal the innate immune responses with PDCoV infection (26) and indicated that weaned piglets might be useful in studying the effect of PEAV on the immune system. However, as we discovered (data not shown), oral delivery of cell-cultured PEAV without a coating didn't cause diseases in weaned piglets. The successful establishment of the infection model in weaned piglets by PEAV-Coated system removed the obstacle. The infection model allows us to control the type and quantity of virus, and to simulate the fecal-oral route, which will help us to better study the pathogenic mechanism of porcine intestinal CoVs and lay a foundation for the preparation of a PEAV oral vaccine.

Innate immunity is thought to be the first line of host defense against a wide variety of pathogenic infections (27). Of note, type I interferon (IFN-α/β), as important cytokines of innate immunity induced by virus invasion, could establish an anti-viral state in infected sites, and also regulate the development of an adaptive immune response (19). The small intestinal mucosa, which contains immune tissues, is thought to be the primary site for defense against enteropathogens (28). Microscopic lesions and viral antigens were also found in the small intestines of PEAV-Coated-challenged piglets (Supplemental Figure 1), indicating that PEAV could destroy mucosal tissue localized in the gut. Peyer's patches, occupied in the jejunum and ileum, serve as the primary inductive sites for intestinal immunity (29–31). It was reported that SADS-CoV antagonizes IFN-β production *via* blocking IPS-1 and RIG-I in IPEC-J2 cells (32). Interestingly, we also found that PEAV could inhibit the mRNA expressions of *IFN-*α and *IFN-*β in Peyer's patches at 7 d.p.i., consistent with the results *in vitro* (Supplemental Figure 2), indicating that PEAV infection could inhibit the body's anti-viral state. It is known that ISGs, such as dsRNA activated protein kinase R (PKR) (33), 2′-5′-oligoadenylate synthetase (OAS) (34), and Mx proteins (35), induced by IFNs can directly act against virus infection (36). Consistent with the type I interferon results, PEAV could inhibit the mRNA expressions of *OAS, Mx1*, and *PKR* in Peyer's patches. All these results suggest that this virus could overcome the antiviral response to infect the body. Interestingly, the antiviral response was not detected *in vivo*, possibly due to the timing of the test. Since PEAV inhibited an antiviral response *in vivo*, several important questions are raised. For example, what's the viral protein of PEAV inhibiting the expressions of these cytokines *in vivo*? And what is the exact underlying mechanism? What's the immune cell dynamics after PEAV infection in pigs? Further efforts will be required to elucidate the molecular mechanisms underlying the pathogenesis of PEAV infection.

In summary, our research successfully established a PEAV animal infection model in weaned piglets. Remarkably, inoculation of weaned piglets with PEAV obviously inhibited *IFN-*α, *IFN-*β, *OAS, Mx1*, and *PKR* mRNA expression in infected Peyer's patches *in vivo*. These findings have provided insights for further studies of the molecular mechanism underlying PEAV infection resistant host immune responses.

## Data Availability Statement

All datasets presented in this study are included in the article/Supplementary Material.

## Ethics Statement

The animal study was supervised by the Institutional Animal Care and Use Committee of Sun Yat-sen University (IACUC DD-17-1003) and used in accordance with regulation and guidelines of this committee.

## Author Contributions

YC and ZX conceived and designed the experiments. ZX and PP performed the experiments. ZX analyzed the data. LG, YL, CX, and YC contributed reagents, materials, analysis tools. ZX wrote the paper. YC checked and finalized the manuscript. All authors read and approved the final manuscript.

## Conflict of Interest

The authors declare that the research was conducted in the absence of any commercial or financial relationships that could be construed as a potential conflict of interest.

## Supplementary Material

Supplemental Figure 1 Intestinal changes in weaned piglets inoculated with PEAV-Coated. **(A,C)** H&E-stained jejunum tissue sections of sucrose microspheres-challenged piglets at 3 d.p.i. and 7 d.p.i. **(B,D)** H&E-stained jejunum tissue sections of PEAV-Coated-challenged piglets at 3 d.p.i. and 7 d.p.i. (Blunt intestinal villus was indicated by arrows). **(E,G)** Immunohistochemically stained jejunum tissue sections of sucrose microspheres-challenged piglets at 3 d.p.i. and 7 d.p.i. **(F,H)** Immunohistochemically stained jejunum tissue sections of PEAV-Coated-challenged piglets at 3 d.p.i. and 7 d.p.i.

Supplemental Figure 2 Infection of IPEC-J2 cells with PEAV strain GDS04 inhibits Sendai virus (SeV)-induced expression of *IFN*-β *in vitro*. IPEC-J2 cells (2 × 10^5^) were mock infected or infected with PEAV at an MOI of 0.5. Twelve hours after PEAV infection, cells were treated with SeV at an MOI of 1. Twelve hours after SeV treatment, mRNA expressions of *IFN*-α **(A)** and *IFN*-β **(B)** were measured by real-time PCR using specific primers. The mRNA expression levels of these molecules were calculated relative to the expression level of *GAPDH*. Data are represented as mean ±SD, *n* = 9. ^*^*p* < 0.05, ^***^*p* < 0.001. (SeV is able to induce a good IFN-β response, but not IFN-α in IPEC-J2 cells).
