# Supplementary figures and images for: Porcine enteric alphacoronavirus Inhibits IFN-α, IFN-β, OAS, Mx1, and PKR mRNA Expression in Infected Peyer's Patches in vivo
Source: Front Vet Sci. 2020 Jul 3;7:449. doi: 10.3389/fvets.2020.00449 (PMC7347908; doi:10.3389/fvets.2020.00449)

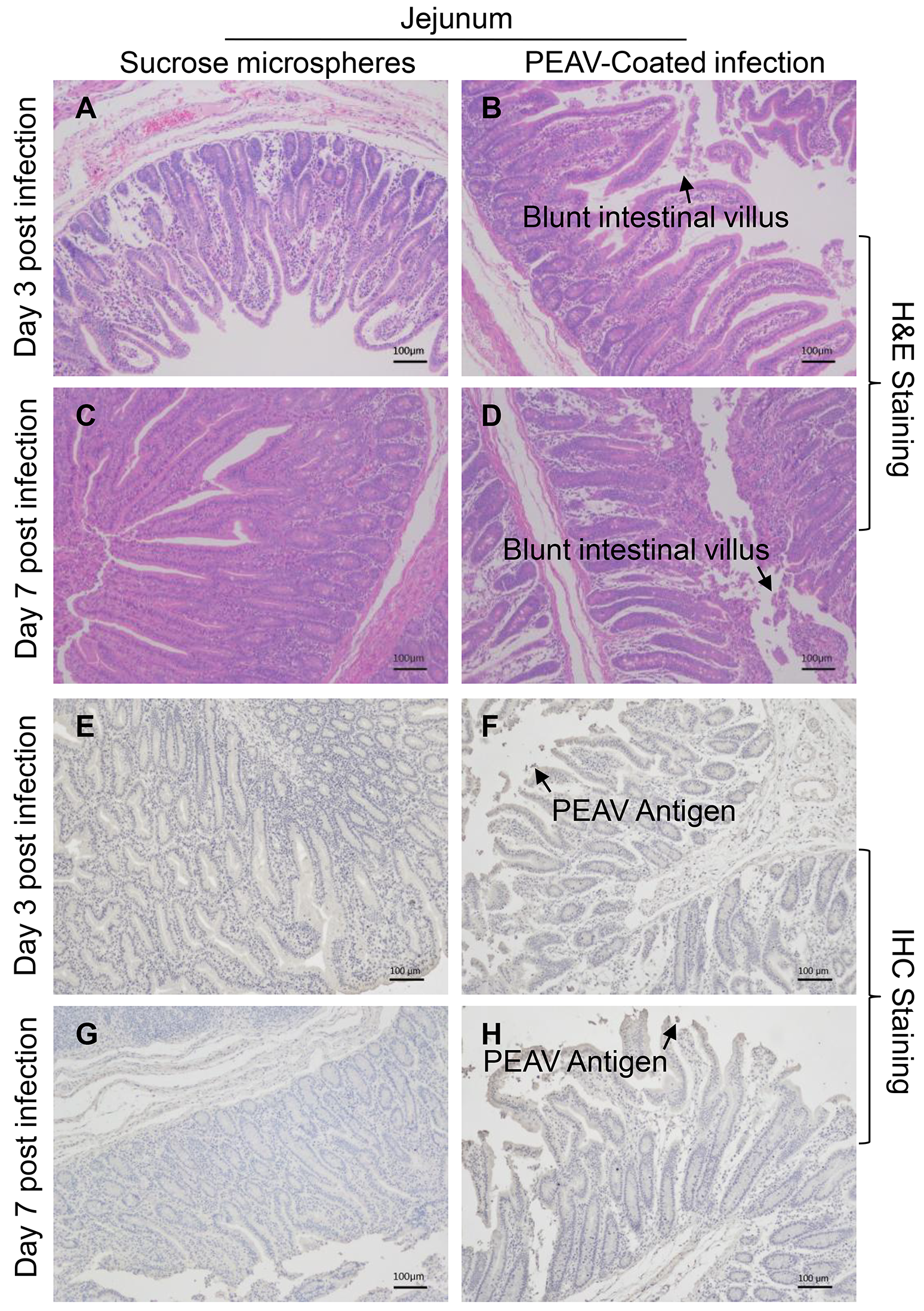

Supplement: Supplemental Figure 1 — Intestinal changes in weaned piglets inoculated with PEAV-Coated. (A,C) H&E-stained jejunum tissue sections of sucrose microspheres-challenged piglets at 3 d.p.i. and 7 d.p.i. (B,D) H&E-stained jejunum tissue sections of PEAV-Coated-challenged piglets at 3 d.p.i. and 7 d.p.i. (Blunt intestinal villus was indicated by arrows). (E,G) Immunohistochemically stained jejunum tissue sections of sucrose microspheres-challenged piglets at 3 d.p.i. and 7 d.p.i. (F,H) Immunohistochemically stained jejunum tissue sections of PEAV-Coated-challenged piglets at 3 d.p.i. and 7 d.p.i. [file Image_1.tif]

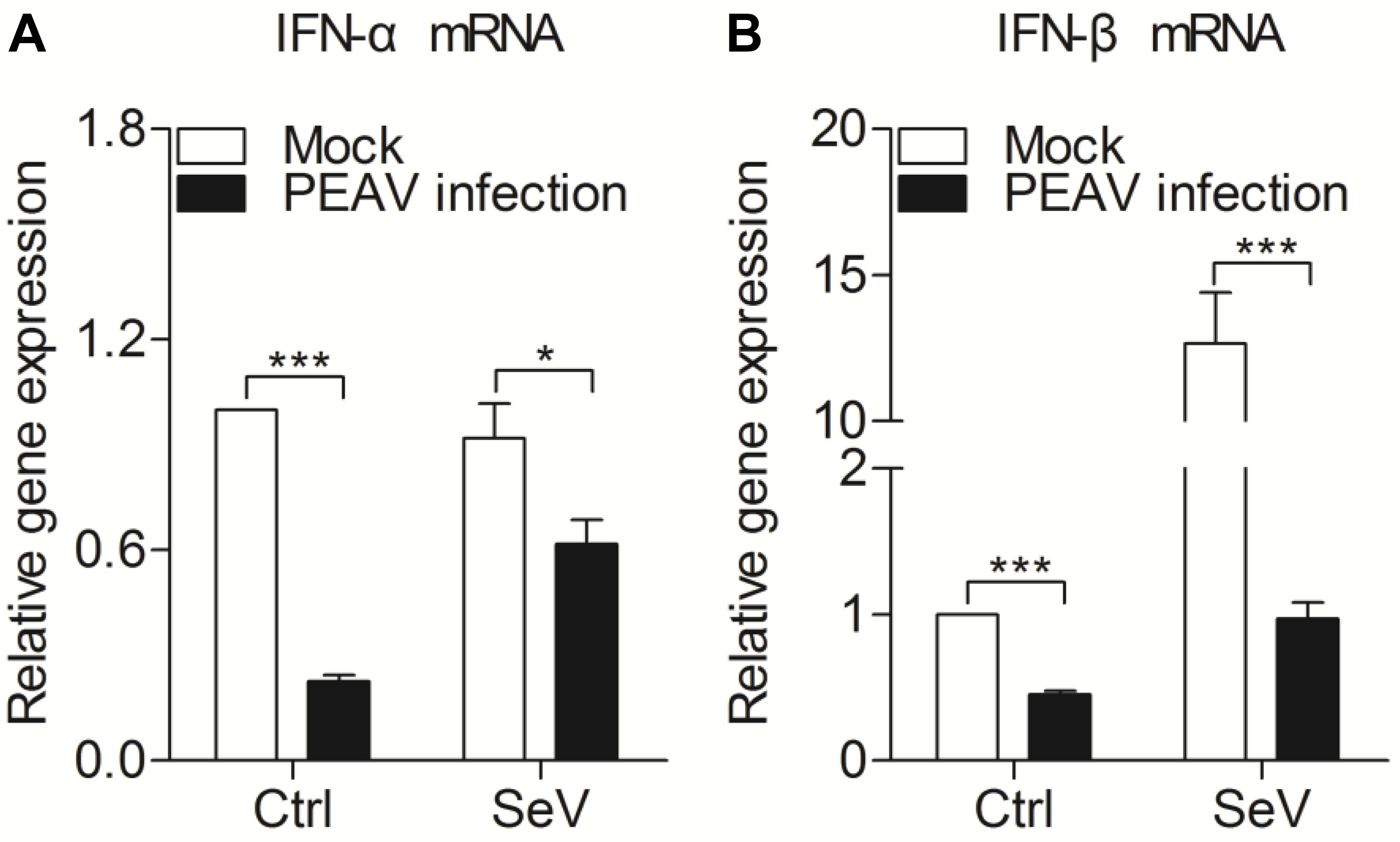

Supplement: Supplemental Figure 2 — Infection of IPEC-J2 cells with PEAV strain GDS04 inhibits Sendai virus (SeV)-induced expression of IFN-β in vitro. IPEC-J2 cells (2 × 10^5^) were mock infected or infected with PEAV at an MOI of 0.5. Twelve hours after PEAV infection, cells were treated with SeV at an MOI of 1. Twelve hours after SeV treatment, mRNA expressions of IFN-α (A) and IFN-β (B) were measured by real-time PCR using specific primers. The mRNA expression levels of these molecules were calculated relative to the expression level of GAPDH. Data are represented as mean ±SD, n = 9. *p < 0.05, ***p < 0.001. (SeV is able to induce a good IFN-β response, but not IFN-α in IPEC-J2 cells). [file Image_2.tif]
